# My Way: development and preliminary evaluation of a novel delivery system for PrEP and other sexual health needs of young women in Western Kenya

**DOI:** 10.1002/jia2.26217

**Published:** 2024-02-20

**Authors:** Jessica E. Haberer, Kevin Oware, Lawrence Juma, Bernard Nyerere, Vincent Momanyi, Josephine Odoyo, Lindsey Garrison, Julita Bhagat, Nicholas Musinguzi, Jared M. Baeten, Aaron Siegler, Elizabeth A. Bukusi

**Affiliations:** ^1^ Massachusetts General Hospital Boston Massachusetts USA; ^2^ Harvard Medical School Boston Massachusetts USA; ^3^ Kenya Medical Research Institute Kisumu Kenya; ^4^ Ark Africa Nairobi Kenya; ^5^ Global Health Collaborative Mbarara Uganda; ^6^ University of Washington Seattle Washington USA; ^7^ Emory University Atlanta Georgia USA

**Keywords:** adherence, adolescent girls and young women, HIV prevention trials, PrEP, point of care, Africa

## Abstract

**Introduction:**

Young women in sub‐Saharan Africa are a priority population for HIV prevention, yet challenges with adherence and persistence to HIV pre‐exposure prophylaxis (PrEP) are common. This study involved the development and pilot testing of My Way—a novel delivery system for PrEP and co‐packaged sexual health services.

**Methods:**

My Way was developed in Kisumu, Kenya through a user‐centred design process (2020). The intervention involves peer‐delivery and support for HIV testing and PrEP use, self‐collected vaginal swabs for sexually transmitted infection (STI) testing, pregnancy testing, oral contraceptive pills, self‐injectable medroxyprogesterone and/or condoms. My Way was assessed among 16‐ to 24‐year‐old sexually active women in a randomized controlled trial versus standard of care (SoC; 2021–2022). Use of PrEP and other sexual health services were tracked at 1, 3 and 6 months for feasibility. Acceptability was determined by questionnaire. The effect of the intervention on tenofovir diphosphate (TFV‐DP) levels was assessed by chi‐square test (primary outcome); other predictors were explored with regression analysis.

**Results:**

Among 150 women, the median age was 22 years and the median number of sexual partners was 2. Moderate/severe depression was common (60%). In the intervention arm, peers made 88% (198/225) of possible kit deliveries (177 with PrEP) and 49 STIs were diagnosed. In the SoC arm, 24% (55/225) of expected clinic visits occurred (53 with PrEP); no STI testing was performed. TVF‐DP was detected in 16 participants at 6 months: 16% (12/75) in the intervention arm versus 5% (4/75) in the SoC arm (*p* = 0.03). Persistence among those with ongoing HIV prevention needs (i.e. prevention‐effective persistence) was 18% (12/67) versus 7% (4/56; *p* = 0.08). No women acquired HIV. The intervention was significantly associated with detectable TFV‐DP (OR 3.5, 1.1‐11.4; *p* = 0.04); moderate/severe depression trended towards an association with TFV‐DP (OR 0.2, 0.03–1.6; *p* = 0.13).

**Conclusions:**

My Way is a promising delivery system for PrEP and other sexual health services among young women in Western Kenya. We found high feasibility and acceptability. PrEP use was modest, but higher with My Way compared to SoC. Long‐acting PrEP formulations may overcome important barriers to PrEP use and should be explored in combination with the My Way delivery model.

## 1 INTRODUCTION

Young women in sub‐Saharan Africa (sSA) are a priority population for HIV prevention, accounting for 63% of all new cases in 2021 [1]. Approximately six of seven new HIV acquisitions occur among adolescents, and young women are twice as likely to be living with HIV than their male counterparts [1]. While daily oral emtricitabine/tenofovir (FTC/TDF) is a highly efficacious means of HIV pre‐exposure prophylaxis (PrEP), numerous trials and PrEP implementation studies among young women in sSA have shown challenges with daily adherence and persistent use over time [2, 3, 4]. Barriers to PrEP adherence are numerous; stigma, logistical challenges in accessing PrEP and low levels of empowerment are especially relevant for persistence [5, 6].

Young women also have many unmet needs for family planning and diagnosis of sexually transmitted infections (STIs). Even when available free‐of‐charge, persistence to multiple forms of family planning is suboptimal [7, 8]. Additionally, STIs are often asymptomatic in women, and the prevalence of STIs has been found to be consistently high in multiple studies screening young women in sSA [3, 9]. Because the standard of care (SoC) for STI management is driven by symptoms, many STIs remain undiagnosed and untreated [10].

These challenges call for innovation in delivering both PrEP and other sexual health services for young women. Community‐based service delivery holds promise to overcome common persistence barriers, including stigma and logistical arrangements [5]. Preliminary work with home‐based delivery has shown the approach to be feasible, acceptable and potentially impactful on PrEP adherence and STI outcomes among men who have sex with men (MSM) in the United States [11]. Similar approaches are being utilized in clinical programmes, including for MSM in Thailand [12], and other approaches to community‐based PrEP delivery are being explored [13]. Evidence for the benefit of delivery to young women in sSA, however, has not been established.

My Way is a novel delivery system designed to meet the HIV prevention and other sexual health needs of young African women. This paper describes the development of My Way, as well as the feasibility, acceptability and preliminary impact of My Way on PrEP use and engagement in sexual health services in a pilot randomized controlled trial.

## 2 METHODS

### 2.1 Setting

This study was conducted in Kisumu, Kenya (HIV prevalence among young women 17.5%) [14]. The two recruitment sites were Lumumba Sub‐County Hospital and Kisumu County Hospital. Study operations were based at the nearby Research Care Training Program that is affiliated with the Kenya Medical Research Institute. PrEP knowledge in the community at the time of the study (2020–2022) was evolving as PrEP became more commonly available through the national HIV prevention programme [15].

### 2.2 Formative phase recruitment (August–December 2020)

Recruitment involved community mobilization for PrEP use among young women in the Kisumu region (e.g. public talks in hotspots, fish landing points and estates with high numbers of young people). Young women aged 16–24 years old without HIV were identified when they presented to the two clinics for PrEP or other sexual health services, using convenience sampling. Other inclusion criteria included sexual activity in the past 3 months, residence in the Kisumu region, phone ownership and ability to understand KiSwahili, DhoLuo and/or English. The only exclusion criterion was the inability to provide consent; pregnancy was not an exclusion criterion. Additional participants, including young men, nurses and community health workers, were identified through snowball sampling as driven by the user‐centred design process.

### 2.3 User‐centred design process

My Way was based on Social Cognitive Theory, in which environmental and cognitive factors are considered as inter‐related influences with behaviour [16]. A Nairobi‐based design firm, Ark Africa, led individual interviews and workshops to develop the intervention. Participants were guided through both ideation and creation processes. Ark Africa designers created prototypes, personas, storyboards and delivery models that they presented to participants for iterative feedback. This process led to refined versions of the kit for optimal feasibility and acceptability.

### 2.4 Kit development

**Figure 1:**
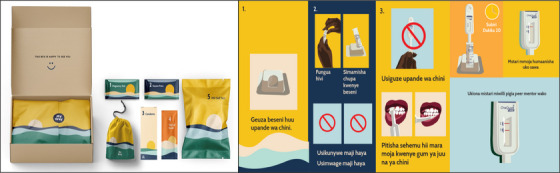
My Way kit and sample instruction pamphlet (kiSwahili). Translation of the kiSwahili: 1. Turn the basin this side down. 2. Open this way. Place the bottle upright on the basin. Do not drink this water. Do not pour this water. 3. Do not turn it upside down. Swab across your gums once, the top gum and the bottom gum. Wait 20 minutes. One line means you are negative. When you see two lines, contact your peer.

Findings gathered during the design process were utilized to construct kits containing HIV self‐testing (choice of oral or fingerstick) and oral PrEP, plus vaginal swabs for self‐sampling to test gonorrhoea and chlamydia, pregnancy testing, oral contraceptive pills, self‐injectable medroxyprogesterone (i.e. Sayana Press) and/or condoms. Sanitary pads were added to enhance appeal. All tests were available for point‐of‐care use in Kenya, and all kit packaging was procured locally in Kisumu. Videos and user‐friendly instructional materials were made for each component of the kit (Figure 1) [17].

### 2.5 Trial recruitment

Participants were recruited directly from Lumumba Sub‐County Hospital or Kisumu County Hospital (July 2021–March 2022) and followed for 6 months. During enrolment, all participants initiating PrEP at the two study sites were approached; PrEP initiation involved HIV, hepatitis B and creatinine testing per Kenya national guidelines [18]. Participants otherwise met the same enrolment criteria as noted above. All participants completed baseline socio‐demographic and behavioural questionnaires and were then randomized 1:1 to the intervention versus the SoC group. Randomization was carried out in variable blocks by the study coordinator and verified via REDCap. Blinding was not possible due to the nature of the intervention.

### 2.6 Intervention arm

Participants in the intervention arm were assigned to peer study staff who were 18–30 years old, had experience taking PrEP and were hired for the study. Peers contacted participants ∼1 week in advance of follow‐up visits at 1, 3 and 6 months at which time the peers delivered the kits. Arrangements for the delivery were individualized and typically involved a peer taking an unmarked motorbike to the participant's preferred location (e.g. home). During the visit, peers supported participants in choosing kit components and ensuring they knew how to use them, utilizing the instructional materials and/or videos developed in the formative phase of the project as needed. HIV and pregnancy testing were always performed prior to providing PrEP and hormonal contraception, respectively. Peers also encouraged participants to contact them via cell phone or WhatsApp for support. Vaginal swabs were brought back to the study clinic for testing (Cepheid). Participants with any positive tests (pregnancy, HIV or STI) received counselling services; all necessary referrals were made for appropriate services. A study physician oversaw care provision and supported peers with clinical questions from participants. To assess acceptability, participants in the My Way arm also completed the System Usability Scale (SUS) and answered questions on preferences and acceptability of the procedures associated with the intervention and use of kit components at 6 months (Table S1) [19]. In‐depth interviews were conducted at the end of participation; those findings will be reported separately.

### 2.7 Standard‐of‐care arm

Participants in the SoC arm were encouraged to follow up with the clinic where they initiated PrEP or another preferred clinic at 1, 3 and 6 months for PrEP refills and other desired sexual health services. After 6 months, clinic records at Lumumba Sub‐County Hospital and Kisumu County Hospital were audited to determine HIV testing, PrEP dispensing, and pregnancy and STI testing; participants self‐reported data about visits to other clinics. Clinic visits were categorized as 1, 3 and 6 months (using the closest time point to the visit date).

### 2.8 Additional procedures

All My Way and SoC participants were asked to come to the study site at 6 months to provide dried blood spots (DBS) for tenofovir diphosphate (TFV‐DP) testing. DBS were sent to Molecular Lab Testing in Vancouver, Washington for analysis by high‐performance liquid chromatography [20, 21].

### 2.9 Analysis

We scored socio‐behavioural scales as follows: problematic alcohol use (RAPS‐4; “yes” to 1+ items was considered positive) [22], depression (PHQ‐9; >9 indicated possible moderate or worse depression) [23], sexual relationship power (scores 1–4 averaged, higher scores indicating less power) [24], intimate partner violence (“yes” to 1+ items indicated violence) [25], HIV stigma (scores 1–4 summed, dichotomized at the median; higher numbers indicated more stigma) [26], PrEP stigma (mean response, 1–5 with reverse coding as needed) [27], self‐esteem (scores 0–3 summed with higher scores indicating more self‐esteem, dichotomized at 15) [28], necessity and concern about PrEP use (modified BMQ; scores 1–5 were averaged, divided into tertiles; higher scores indicated stronger agreement with necessity or concern for PrEP) [29], clinic satisfaction (16‐items related to care, respect, problem‐solving, choices, waiting times, support and coping; not a standardized or validated scale) and SUS (score summed for each item, normalized to a scale of 0–100) [30].

We pre‐specified feasibility as delivery of >75% of My Way kits to participants when desired at months 1, 3 and 6. We also pre‐defined acceptability as an SUS score indicating good or better performance (i.e. >60). The effect of the intervention on PrEP use was first determined in an intention‐to‐treat manner (i.e. all participants enrolled in the trial in their assigned arm, regardless of ongoing HIV prevention needs) using a chi‐square test of detectable versus undetectable TFV‐DP (threshold of 100 fmol/punch) in the two study arms; missing values were considered undetectable. We also explored prevention‐effective persistence by only considering participants who reported ongoing HIV prevention needs [31]. No consensus exists on the definition of HIV prevention needs. In our analysis, we included participants with 1 or more of the following: (1) PrEP use (per self‐report and/or detectable TFV‐DP); (2) >1 concurrent sexual partner; (3) condomless sex; and (4) indication of “some/a lot of concern about getting HIV” at month 6 (data at months 1 and 3 were limited to self‐report in the intervention arm only). Univariable logistic regression analysis was used to assess for predictors of detectable TFV‐DP. We evaluated the demographic and behavioural factors noted above with the highest potential for significant impact based on the literature [3, 4, 5]. Given the small sample size, we did not perform multivariable modelling. We used STATA (version 5.1) to perform all analyses; a *p*‐value of <0.05 was considered statistically significant for all analyses.

### 2.10 Ethics and reporting

All participants provided written informed consent. Caregivers for those 16–17 years old also provided assent. All study procedures were approved by the institutional review boards at the Kenya Medical Research Institute (KEMRI) and MassGeneral Brigham. Trial findings are presented according to CONSORT guidelines [32].

## 3 RESULTS

### 3.1 User‐centred design process

The formative work to develop the My Way intervention involved 40 young women interested in taking PrEP, 7 young women who could serve as peers to deliver PrEP, 16 young men and 6 health professionals (nurses, a lab technician and community health workers). All individuals approached for participation were eligible and agreed to enrol.

Key design opportunities included visual appeal, stigma around PrEP use, privacy and confidentiality concerns, illiteracy and communication issues, and peer mentor training needs. Solutions led to early prototypes for the My Way kit (e.g. using non‐descript external packaging but attractive internal component designs) and associated educational materials for each kit component (i.e. easy‐to‐use pamphlets and videos), as well as approaches to community sensitization and engagement. Priority was placed on presenting information simply and clearly with peer support. Peers were identified as the central touchpoint for the intervention, warranting careful training and support. Mobile phone communication was seen as important to individualize kit components, arrange delivery, provide support and answer questions. See Figure 1 for the final kit components and a sample pamphlet.

### 3.2 Trial participant characteristics

**Figure 2:**
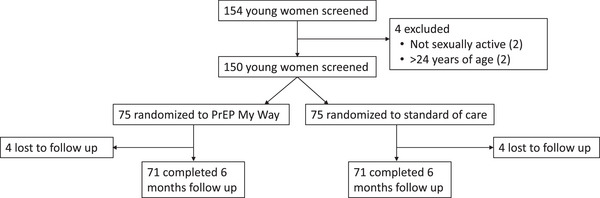
Participant flow diagram.

A total of 154 women were screened for trial participation, of whom 150 were enrolled (Figure 2). Reasons for non‐enrolment were not being sexually active in the past 3 months (*N* = 2), and >24 years of age (*N* = 2); all exclusions took place prior to randomization. Four participants in the My Way arm and four participants in the SoC arm were lost to follow‐up. Median duration of follow‐up was 5.7 months (2.3, 6.8) and 5.7 months (2.3, 7.4), respectively. Ongoing HIV prevention need at 6 months was seen in 67 participants in the My Way arm versus 56 participants in the SoC arm.

As shown in Table 1, participants in the two arms were largely similar across all baseline characteristics. The median age was ∼22 years; none was pregnant. About half were students, while most others were unemployed or had unsteady employment. The majority were living with parents or other family, although slightly more in the SoC arm were living alone (19% vs. 13%); just over 10% were living with their sexual partners in both arms. Somewhat more participants in the My Way arm were single with a steady partner (48% vs. 35%) with relatively more in the SoC arm being single with no steady partner (51% vs. 44%) and married as a single wife (11% vs. 5%). Most participants lived in semi‐urban or urban areas and travelled <1 hour to the clinic. About one‐fifth reported a chronic medical condition and ∼40% had previously used a daily medication. Some degree of food insecurity was common.

**Table 1:** Baseline socio‐behaviour characteristics of young women in the trial

Characteristic	My Way (*N* = 75)	Standard of care (*N* = 75)
Median age in years (IQR)	21.6 (20.7, 23.1)	22.6 (20.9, 23.9)
Median years of education (IQR)	12 (12, 14)	12 (12, 14)
Able to read (*N*, %)	75 (100%)	75 (100%)
Job/occupation (*N*, %)		
Student	41 (55%)	38 (51%)
Employment (steady)	1 (1%)	0 (0%)
Employment (not steady)	19 (25%)	18 (24%)
Parent of child	0 (0%)	1 (1%)
Household help	0 (0%)	0 (0%)
Unemployed	13 (17%)	16 (21%)
Other	1 (1%)	2 (3%)
Living status (*N*, %)		
With parents	37 (49%)	28 (38%)
With other family	18 (24%)	17 (23%)
With a sexual partner	8 (11%)	9 (12%)
With friends	2 (3%)	2 (3%)
Alone	10 (13%)	14 (19%)
Other	0 (0%)	3 (4%)
Marital status (*N*, %)		
Single (no steady partner)	33 (44%)	38 (51%)
Single (steady partner)	36 (48%)	26 (35%)
Married (1 wife)	4 (5%)	8 (11%)
Married (multiple wives)	2 (3%)	2 (3%)
Widowed/divorced/separated	0 (0%)	1 (1%)
Residence (*N*, %)		
Rural	9 (12%)	7 (9%)
Semi‐urban	24 (32%)	22 (29%)
Urban	42 (56%)	46 (61%)
Time to clinic (*N*, %)		
<30 minutes	19 (25%)	23 (31%)
30–60 minutes	52 (69%)	50 (67%)
>60 minutes	4 (5%)	2 (3%)
Chronic medical conditions (*N* yes, %)	14 (19%)	11 (15%)
Prior use of daily medication (*N* yes, %)	32 (43%)	31 (41%)
Not enough food on most days (*N*, %)		
Never	33 (44%)	39 (52%)
Sometimes	40 (53%)	29 (39%)
Often	2 (3%)	7 (9%)

Table 2 presents behaviours potentially associated with HIV prevention needs and/or PrEP use, which were also largely similar between the two study arms. The median number of current sexual partners was 1 (main) and 1 (casual). The HIV status of most partners was unknown. Participants reported having sex a median of three times in the past month, just under half with any condom use. Any transactional sex was slightly higher in the My Way arm (64% vs. 49% in the SoC arm). Problem alcohol use was around 10%, whereas moderate or severe depression was common at ∼60%. Sexual relationship power was moderate; intimate partner violence was somewhat higher in the SoC arm (16% vs. 5% in the My Way arm). Stigma against HIV and PrEP were moderate. Nearly, all participants felt that PrEP prevents HIV, and most felt PrEP makes sex completely safe from HIV. The perceived necessity for PrEP was generally high, whereas concerns about taking PrEP were more evenly distributed. Clinic satisfaction was high and finding time to get to clinic was not difficult for most participants (slightly higher in the My Way arm at 72% vs. 60% in the SoC arm).

**Table 2:** Baseline behaviours among young women with potential association for HIV prevention need and/or PrEP use

Characteristic	My Way (*N* = 75)	Standard of care (*N* = 75)
History of an STI (*N* yes, %)	4 (5%)	6 (8%)
Total median *N* of current sexual partners (IQR)		
Main	1 (1, 1)	1 (1, 1)
Casual	1 (0, 2)	1 (0, 2)
Any partner with HIV		
Yes	3 (4%)	4 (5%)
No	15 (20%)	21 (28%)
Don't know	57 (76%)	50 (67%)
Sexual behaviour		
Median times in the past month (IQR)	3 (1, 5)	3 (1, 6)
Any reported condom use (*N*, %)	26 (49%)	24 (42%)
Any transactional sex (*N*, %)	48 (64%)	37 (49%)
Problem alcohol use, RAPS‐4 (*N*, %)	9 (12%)	7 (9%)
Moderate or severe depression symptoms, PHQ‐9 (*N*, %)	42 (56%)	48 (64%)
Median sexual relationship power score (IQR; possible range 1–4)	2.7 (2.5, 3.0)	2.6 (2.3, 3.0)
Intimate partner violence (*N*, %)	4 (5%)	12 (16%)
Median HIV stigma score (IQR; possible range 1–4)	3.0 (2.3, 3.0)	3.0 (2.3, 3.0)
Median PrEP stigma score (IQR; possible range 1–5)	3.5 (3.1, 3.9)	3.5 (3.2, 3.9)
PrEP beliefs		
PrEP can prevent you from getting HIV		
Yes	74 (99%)	72 (96%)
No	1 (1%)	3 (4%)
Maybe	0 (0%)	0 (0%)
PrEP makes sex completely safe from HIV		
Yes	59 (79%)	57 (76%)
No	7 (9%)	4 (5%)
Maybe	2 (12%)	14 (19%)
Perceived necessity for PrEP (tertiles)		
Low (1–1.3)	12 (16%)	12 (16%)
Moderate (1.5–1.8)	24 (32%)	20 (27%)
High (2–4.3)	39 (52%)	43 (57%)
Perceived concern about taking PrEP (tertiles)		
Low (1.3–3.5)	26 (35%)	23 (31%)
Moderate 3.6–3.9)	24 (32%)	32 (43%)
High (4–5)	25 (33%)	20 (27%)
Clinic satisfaction (median; IQR, possible range 1–4)	3.6 (3.2, 3.9)	3.5 (3.2, 3.9)
Difficulty in finding time to get to clinic		
No problem	54 (72%)	45 (60%)
Somewhat difficult	18 (24%)	27 (36%)
Very difficult	3 (4%)	3 (4%)

### 3.3 Feasibility of the My Way intervention

**Figure 3:**
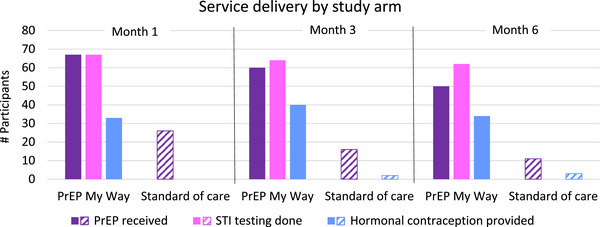
PrEP delivery and sexually transmitted infection testing by study arm. No STI testing occurred in the standard of care arm.

Overall, 198/225 (88%) possible kit deliveries were successfully made among 70 of 75 participants in the My Way arm: month 1 (*N* = 70), month 3 (*N* = 66) and month 6 (*N* = 62). Kit delivery exceeded the predefined threshold for the feasibility of 75% at all time points. As shown in Figure 3, participants requested PrEP and STI testing in most deliveries. While PrEP delivery declined over time, it remained high in young women who indicated ongoing HIV prevention needs and received kits: month 1 (67/70; 96%), month 3 (60/66; 91%) and month 6 (50/62; 81%). A total of 177 deliveries included PrEP and 49 STIs were diagnosed among 30 unique participants (40 chlamydia, 9 gonorrhoea). Pregnancy testing was performed at all kit deliveries with four positive tests. Fourteen participants had contraceptive implants at enrolment; other hormonal contraception options were provided for 53 unique participants with oral contraceptives provided in 79 kits and self‐injectable medroxyprogesterone administered 28 times. Condoms were distributed to 66 unique participants in 163 kits.

In contrast in the SoC arm, 55/225 (24%) possible follow‐up clinic visits occurred in 29 participants (range of 1–3 visits) and decreased over time: month 1 (*N* = 28), month 3 (*N* = 16) and month 6 (*N* = 11). A total of 53 visits involved PrEP dispensing, and no STI testing was performed at any visits. Four young women tested positive for pregnancy in the clinic. Four participants had intrauterine devices and 19 had contraceptive implants at enrolment. Injectable medroxyprogesterone was provided five times during follow‐up.

### 3.4 Acceptability

The median SUS for the 68 (91%) participants in the My Way arm who completed the survey at 6 months was 76 (interquartile range [IQR] 68–85); an SUS >60 indicates a good or better performance. Thus, our findings exceeded our predefined threshold for acceptability. Considering all 75 intervention participants, 66 (88%) reported preferring the intervention to clinic‐based care, and 68 (91%) would use it again in the next year; 69 (92%) found clinic initiation of PrEP, and 69 (92%) found meeting up with the peer to be acceptable or highly acceptable. Between 95% and 99% of participants using various components and aspects of the intervention (HIV testing, self‐injection medroxyprogesterone, vaginal swabs, follow‐up in clinic for any infections or other problems) found them acceptable or very acceptable (Table S1).

### 3.5 Impact on PrEP use at 6 months

TFV‐DP levels were available for 139 participants (69 in the My Way arm, 70 in the SoC arm). The overall median TFV‐DP level was 239 fmol/punch among those with detectable TFV‐DP: 215 fmol/punch (range 100–1919 fmol/punch) in the My Way arm and 772 fmol/punch (range 195–3229 fmol/punch) in the SoC arm. TVF‐DP was detected in 16 participants (12/75 [16%] in the My Way arm vs. 4/75 [5%] in the SoC arm) (*p* = 0.04, intention‐to‐treat approach persistence). Prevention‐effective persistence was 18% (12/67) in the My Way arm versus 7% (4/56) in the SoC arm (*p* = 0.08). No participants acquired HIV.

As shown in Table S2, assignment to the intervention (OR 3.5, 1.1–11.4; *p* = 0.04) was the only factor associated with detectable TFV‐DP, although the presence of moderate/severe depression symptoms trended towards an effect (OR 0.2, 0.03, 1.6; *p* = 0.13). Non‐significant factors included age, student status, problem alcohol use, number of current sexual partners, transactional sex, having an STI (during follow‐up), condomless sex, sexual relationship power, intimate partner violence, HIV stigma, PrEP stigma, perceived necessity of PrEP, perceived concern about PrEP and difficulty getting to clinic.

## 4 DISCUSSION

Through a user‐centred design process, we developed the My Way intervention to support the persistence of PrEP and other sexual health services among young women in Kenya. The intervention focuses on identified needs for appeal, privacy and confidentiality, overcoming illiteracy and communication barriers, and highlighting the key role of the peer. In our pilot trial, we found high feasibility of delivering My Way kits as planned versus few clinic visits in the SoC arm over the same period. PrEP, STI testing, pregnancy testing and contraceptive options were highly utilized in the intervention arm, and acceptability was high with an excellent SUS score and most participants preferring My Way to SoC. The number of young women using PrEP at 6 months per detectable TFV‐DP was modest, but higher in the My Way arm compared to SoC by intention‐to‐treat and prevention‐effective persistence analyses. Notably, the median level of TFV‐DP among those with detectable drug levels was higher in the SoC arm, which may reflect high motivation for PrEP use among the relatively few women who were able to use clinic‐based services.

The high feasibility and acceptability of My Way speak to its potential to reach young women for HIV prevention and other sexual health services. Many HIV prevention studies with this population have shown rapid disengagement from care, often within 1–3 months [2, 3, 4]. Similar experience has also been seen with engagement in other sexual health services, such as family planning. For example, within 1 year, as few as half of young women consistently receive hormonal contraception injections at 1 year, raising concerns about ultimate impact in practice [33]. Clinicians, researchers and public health experts have long called for services to meet clients where they are, particularly for youth who often have other developmentally expected priorities (e.g. basic needs and pleasure) [34]. Community‐based care delivery is gaining momentum with success seen for targeted programmes, like HIV testing and linkage to care [13, 35]. My Way builds on this premise by optimizing end‐user preferences and combining numerous desired services that are safe, currently available and readily delivered at the point‐of‐care. The low clinic‐based utilization of PrEP, STI testing and contraception in our study highlights the importance of this alternative delivery model.

The modest level of PrEP use at 6 months, however, indicates that important additional barriers remain despite this highly feasible and acceptable peer‐supported, community‐delivered approach. As detailed elsewhere [5, 6], PrEP adherence and persistence can be influenced by numerous individual‐level factors (e.g. depression, substance use, side effects) and community factors (e.g. need for social support). Notably, over half of our participants reported moderate or severe depression symptoms, which trended towards a negative association with detectable TFV‐DP. Additionally, two of the key hypothesized barriers that My Way can overcome—clinic dissatisfaction and difficulty getting to clinic—were reported by a minority of young women in our recruited population. The intervention may, therefore, perform better in populations for whom these concerns play a greater role in PrEP use.

Importantly, our assessment of PrEP use by TFV‐DP levels was limited to the 6‐month time point by cost. PrEP use may have been higher earlier in the study when the need for PrEP was also higher. Indeed, PrEP use is only necessary when an individual has need for HIV prevention (i.e. prevention‐effective adherence and persistence) [31]. Our prior qualitative research with young women in Kenya revealed that participation in PrEP programmes can result in behaviour change such that the risk of HIV exposure becomes quite low. For example, many young women reported feeling empowered to reduce the number of sexual partners and increase condom use [36]. This principle of prevention‐effective adherence and persistence reflects rational PrEP use, although the best approach to assessing the ongoing HIV prevention needs is unclear [37, 38]. We used several definitions based on our experience and the literature [5]; however, future studies should explore this important concept.

This study is limited to a single region and small sample size; results should thus be interpreted as preliminary. Selection bias may also have been present due to potentially above‐average community awareness and engagement in HIV prevention services in the study setting. Additionally, the study was conducted during the COVID‐19 pandemic, which may have impacted study engagement. and receipt of services may not indicate actual use of services. The study also has multiple strengths, including a robust user‐centred design process, randomized trial design, and focus on feasibility and acceptability.

## 5 CONCLUSIONS

My Way is a promising delivery system for PrEP and other sexual health services for the high‐priority population of young women in Kenya and potentially elsewhere in sSA. Its high degree of feasibility demonstrates potential for scalability in this context and the excellent acceptability metrics indicate how this intervention meets many needs of this population. Long‐acting PrEP formulations, such as injections or vaginal rings, have been shown to be beneficial for HIV prevention and may overcome some of the remaining barriers to PrEP use [39, 40]. These formulations could readily be introduced into the My Way intervention, although additional research would be needed to explore differences in the delivery of each formulation. Moreover, additional support may be needed for depression. Such an innovative combination of desired products and services holds promise for addressing the critical HIV prevention needs among young women.

## COMPETING INTERESTS

JEH has been a paid consultant for Merck. JMB is employed by Gilead Sciences, outside of the present work.

## AUTHORS’ CONTRIBUTIONS

JEH, JMB, AS and EAB conceived of the study. KO, LJ, BN, VM, JO, LG and JB implemented the study. NM performed statistical analysis. JEH wrote the first version of the manuscript. All other authors contributed to and approved the final version of the manuscript.

## FUNDING

The study was supported by the US National Institutes of Health (R34MH122362). Cepheid donated gonorrhoea and chlamydia test kits.

## Supporting information


**Appendix Table 1**. Acceptability of using various components of My Way. Percentages reflect the total number of participants who reported using the given component at some point during the 6 months of follow‐up
**Appendix Table 2**. Potential socio‐behavioral factors as predictors of detectable (vs undetectable) tenofovir diphosphate levels at 6 months. Values reflect univariable regression analysis controlling for randomization

## Data Availability

Once this manuscript has been published, all quantitative study data will be deidentified and posted for analysis on the Harvard Dataverse.
