# Supplementary material for: My Way: development and preliminary evaluation of a novel delivery system for PrEP and other sexual health needs of young women in Western Kenya
Source: J Int AIDS Soc. 2024 Feb 20;27(2):e26217. doi: 10.1002/jia2.26217 (PMC10879470; doi:10.1002/jia2.26217)
Supplement: Supplementary file 1 — Appendix Table 1. Acceptability of using various components of My Way. Percentages reflect the total number of participants who reported using the given component at some point during the 6 months of follow‐up Appendix Table 2. Potential socio‐behavioral factors as predictors of detectable (vs undetectable) tenofovir diphosphate levels at 6 months. Values reflect univariable regression analysis controlling for randomization [file JIA2-27-e26217-s001.docx]

Appendix Table 1. Acceptability of using various components of My Way. Percentages reflect the total number of participants who reported using the given component at some point during the 6 months of follow-up.

|  | N | Very Acceptable | Acceptable | Neutral | Unacceptable | Very Unacceptable |
| --- | --- | --- | --- | --- | --- | --- |
| 1. Using the HIV self-test kit | 67 | 51  (76%) | 14  (20%) | 1  (2%) | 0  (0%) | 1  (2%) |
| 2. Using the pregnancy test | 67 | 51  (76%) | 15  (22%) | 0  (0%) | 0  (0%) | 1  (2%) |
| 3. Using the self-injection medroxyprogesterone | 22 | 13  (59%) | 8  (36%) | 1  (5%) | 0  (0%) | 0  (0%) |
| 4. Doing the vaginal swab for STIs | 67 | 45  (68%) | 19  (28%) | 1  (2%) | 0  (0%) | 2  (3%) |
| 5. Following up with the clinic for any infections or other problems | 45 | 29  (65%) | 15  (33%) | 0  (0%) | 0  (0%) | 1  (2%) |

Appendix Table 2. Potential socio-behavioral factors as predictors of detectable (vs undetectable) tenofovir diphosphate levels at 6 months. Values reflect univariable regression analysis controlling for randomization.

| Socio-behavioral factor | N (%) or  median (IQR) | OR | 95% CI | p-value |
| --- | --- | --- | --- | --- |
| Study arm  Control  Intervention (My Way) | 139  69 (50)  70 (50) | ref  3.5 | 1.1 - 11.4 | 0.04 |
| Age (years) | 22.3 (20.8-23.6) | 1.2 | 0.9 - 1.5 | 0.33 |
| Student (vs other job/occupation) | 69 (50) | 1.6 | 0.6 - 4.8 | 0.38 |
| Problem alcohol use (RAPS-4) | 9 (6) | 1.2 | 0.1 - 10.8 | 0.9 |
| Depression symptoms (PHQ-9)  Mild  Moderate/severe | 185 (76)  34 (24) | ref  0.2 | 0.03 - 1.6 | 0.13 |
| Number of current sexual partners | 1 (1,1) | 1.0 | 0.6 - 1.8 | 0.98 |
| Transactional sex | 21 (15) | 1.2 | 0.3 - 0.4 | 0.84 |
| Having a sexual transmitted infection | 14 (10) | 0.7 | 0.2 - 3.7 | 0.69 |
| Condomless sex | 86 (62) | 0.8 | 0.3 - 2.6 | 0.77 |
| Sexual relationship power score (per 1 point) increase) | 3 (2.7-3.9) | 1.7 | 0.7 - 4.0 | 0.26 |
| Intimate partner violence* | - | -- | -- | -- |
| HIV stigma score (per 5 units; possible range 4-16) | 13 (11-16) | 1.0 | 0.4 - 2.2 | 0.97 |
| PrEP stigma score (per 5 units; possible range 5-65) | 49 (44-57) | 0.9 | 0.7 - 1.3 | 0.62 |
| Perceived necessity for PrEP  Least  Moderate  Most | 44 (32)  38 (27)  57 (41) | ref  1.7  0.8 | 0.5 - 6.4  0.2 - 3.1 | 0.52 |
| Perceived concern about taking PrEP  Least  Moderate  Most | 26 (19)  41 (30)  72 (52) | ref  0.6  1.1 | 0.1 - 3.1  0.3 - 4.5 | 0.61 |
| Difficulty finding time to get to clinic | 46 (33) | 0.6 | 0.2 - 2.1 | 0.45 |

*Model did not converge due to limited data
